# Simple Scoring System and Artificial Neural Network for Knee Osteoarthritis Risk Prediction: A Cross-Sectional Study

**DOI:** 10.1371/journal.pone.0148724

**Published:** 2016-02-09

**Authors:** Tae Keun Yoo, Deok Won Kim, Soo Beom Choi, Ein Oh, Jee Soo Park

**Affiliations:** 1 Department of Ophthalmology, Yonsei University College of Medicine, Seoul, Republic of Korea; 2 Department of Medical Engineering, Yonsei University College of Medicine, Seoul, Republic of Korea; 3 Graduate Program in Biomedical Engineering, Yonsei University, Seoul, Republic of Korea; 4 Department of Medicine, Yonsei University College of Medicine, Seoul, Republic of Korea; 5 Department of Anaesthesiology and Pain Medicine, Yonsei University College of Medicine, Seoul, Republic of Korea; Instituto Nacional de Ciencias Medicas y Nutricion Salvador Zubiran, MEXICO

## Abstract

**Background:**

Knee osteoarthritis (OA) is the most common joint disease of adults worldwide. Since the treatments for advanced radiographic knee OA are limited, clinicians face a significant challenge of identifying patients who are at high risk of OA in a timely and appropriate way. Therefore, we developed a simple self-assessment scoring system and an improved artificial neural network (ANN) model for knee OA.

**Methods:**

The Fifth Korea National Health and Nutrition Examination Surveys (KNHANES V-1) data were used to develop a scoring system and ANN for radiographic knee OA. A logistic regression analysis was used to determine the predictors of the scoring system. The ANN was constructed using 1777 participants and validated internally on 888 participants in the KNHANES V-1. The predictors of the scoring system were selected as the inputs of the ANN. External validation was performed using 4731 participants in the Osteoarthritis Initiative (OAI). Area under the curve (AUC) of the receiver operating characteristic was calculated to compare the prediction models.

**Results:**

The scoring system and ANN were built using the independent predictors including sex, age, body mass index, educational status, hypertension, moderate physical activity, and knee pain. In the internal validation, both scoring system and ANN predicted radiographic knee OA (AUC 0.73 versus 0.81, *p*<0.001) and symptomatic knee OA (AUC 0.88 versus 0.94, *p*<0.001) with good discriminative ability. In the external validation, both scoring system and ANN showed lower discriminative ability in predicting radiographic knee OA (AUC 0.62 versus 0.67, *p*<0.001) and symptomatic knee OA (AUC 0.70 versus 0.76, *p*<0.001).

**Conclusions:**

The self-assessment scoring system may be useful for identifying the adults at high risk for knee OA. The performance of the scoring system is improved significantly by the ANN. We provided an ANN calculator to simply predict the knee OA risk.

## Introduction

Knee osteoarthritis (OA) is the most common joint disease of adults worldwide [1]. Since there has been no effective disease-modifying therapy, the treatments of advanced radiographic knee OA are limited [2]. Studies have shown that the early diagnosis and treatment of OA could help to prevent aggravation of symptoms [3], [4]. Late diagnosis results in the socio-economic burden of illness associated with OA. Therefore, clinicians face a significant challenge of identifying patients who are at high risk of radiographic and symptomatic OA in a timely and appropriate way [4].

Early detection of knee OA can be assisted by knowledge of risk factors. The risk factors of knee OA are well-known and include female, older age, obesity, knee injury, and occupational factors [5]. To evaluate the relationship between risk factors and knee OA, several methods have been proposed. A screening questionnaire for symptomatic knee OA was developed based on patients' self-reported symptoms [6]. However, this screening tool showed low specificity, and could not predict radiographic knee OA without pain. A logistic regression (LR) model was also developed using well-recognized risk factors such as age, sex, body mass index, occupational factor, and joint injury [7]. This risk prediction model needs calculating a complicated LR equation. Other algorithms have been based on the combination of clinical information including physical examination, blood examination for specific molecules, and genetic data [4], [5], [8]. However, these prediction models were inefficient due to the low performance. In addition, their risk prediction models were not convenient for the laypersons or clinicians. Consequently, it is warranted to develop new simple and convenient prediction tools to identify patients at high risk for knee OA.

Artificial neural network (ANN) is an area of artificial intelligence technology and a mathematical system which mimic biological neural networks [9]. The networks can be trained to recognize underlying patterns of diseases. Once appropriate training is performed, the neural networks attempt to predict with a higher accuracy than conventional classification analysis. Until recently, there have been many advances in methodology of ANN to find an optimal predictive model automatically [10], [11]. Due to the ability to detect complex nonlinear relationships between predictors and diseases, ANN has been successfully used in medical decision support systems [12], [13].

This study aimed at the first development and validation of a new self-assessment scoring system and ANN for radiographic and symptomatic knee OA risk prediction. We developed a scoring system for knee OA prediction based on simple surveys from large population dataset, which can be easily calculated. ANN analysis was performed to improve the scoring system for knee OA risk prediction. No reports have investigated the ability of ANN in a clinical manner for knee OA risk prediction. For convenient use, we provided a simple ANN calculator for predicting radiographic and symptomatic knee OA.

## Participants and Methods

This cross-sectional study investigated the prediction models for incidence of radiographic and symptomatic knee OA. All analyses were based on the Fifth Korean National Health and Nutrition Examination Survey (KNHANES V-1, online at http://knhanes.cdc.go.kr/knhanes) and the Osteoarthritis Initiative (OAI, online at www.oai.ucsf.edu). Since all data were available on the web and data analysis was secondary, no ethical statement was required for this work. The KNHANES V-1 was approved by the institutional review board (IRB) of the Korean Centers for Disease Control and Prevention (approval no. 2010-02CON-21-C), and all participants provided written consent. The OAI was approved by the IRB for the Committee on Human Research, University of California, San Francisco (approval no. 10–00532).

### Data source and subjects

The KNHANES is an ongoing population-based and nationwide epidemiological survey conducted by the Korea Center for Disease Control and Prevention, Ministry of Health and Welfare [14]. The KNHANES consists of a health interview survey, a health examination survey (physical examination and clinical measurements), and a nutrition survey. In the KNHANES V-1 conducted in 2010, bilateral knee plain radiographs were assessed for all participants older than 50 years. All individuals, total participants from 3840 households, were randomly selected from 192 survey locations using stratified sampling, considering population gender, age, regional area, and type of residential area.

Initial candidates for this study included 3075 participants. Eligible participants were those who underwent both the right and left knee radiographic examination. To reduce the confounding factors that might influence knee OA, we excluded 315 participants who were receiving treatment for knee OA. We also excluded 16 participants who did not respond to the medical history interview and 79 participants with missing data in the health examination survey. Finally, a total of 2665 participants were included in this study.

**Fig 1:**
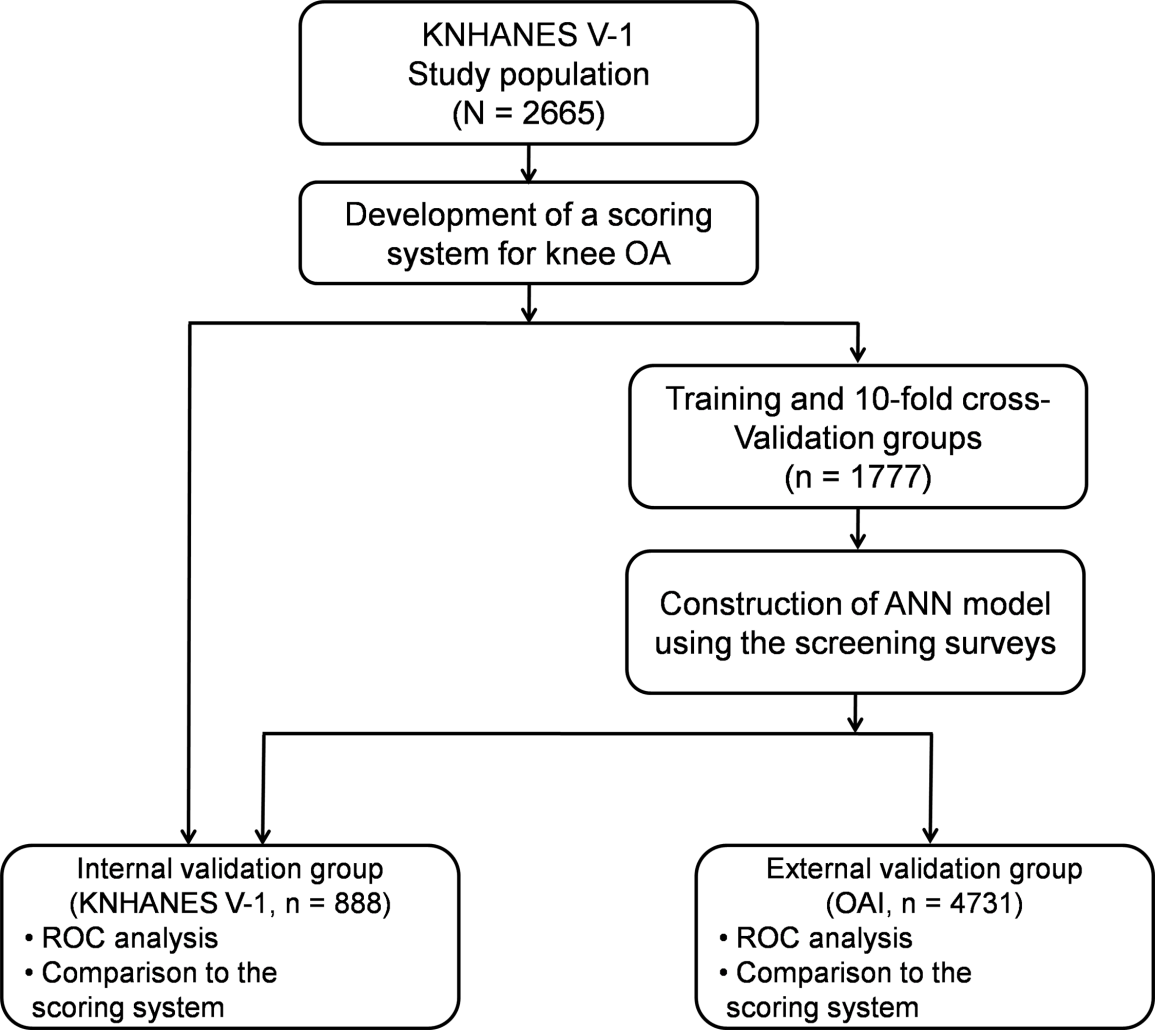
Dataset used in the development and validation for knee osteoarthritis risk prediction. ANN, artificial neural network; KNHANES, Korea National Health and Nutrition Examination Survey; OA, osteoarthritis; OAI, Osteoarthritis Initiative; ROC, receiver operating characteristic.

The dataset were separated randomly into two independent groups, training and internal validation groups (Fig 1). The training group, comprised of two thirds (1777 participants) of the entire dataset, was used to construct an ANN model. The internal validation group, comprised of one third (888 participants) of the entire dataset, was used to assess the ability to predict knee OA.

### Health interview survey and physical examination

The health interview survey, including knee OA symptoms, was conducted through a face-to-face interview by trained interviewers. In the KNHANES V-1, we defined participants as having knee pain or stiffness by asking whether they had experienced knee pain or stiffness for more than 30 days during last 3 months. Each participant was also interviewed and completed a questionnaire exploring educational status, household income, alcohol consumption, smoking status, diabetes mellitus, hypertension, and physical activities. Walking, moderate, and heavy physical activities were measured as the average time per day. Hypertension was defined as a diastolic blood pressure (DBP) ≥ 90 mmHg, a systolic blood pressure (SBP) ≥ 140 mmHg, a self-reported physician diagnosis, or use of anti-hypertensive medications [15]. Moderate activities included activities such as carrying light objects, sweeping, mopping, vacuuming, and brisk walking. Heavy activities included occupational works involving heavy lifting and strenuous sports or recreation. Height, weight, and waist circumference were measured, and body mass index (BMI) was calculated.

### Radiographic examination of the knee and definition of knee OA

In the KNHANES V-1, bilateral anteroposterior, lateral, and weight-bearing anteroposterior plain radiographs of knees were taken [16]. Radiographic changes relating to OA were assessed using the Kellgren/Lawrence (KL) grade [17]. The radiographic images were graded by trained two radiologists with concordant grades accepted. When there was a difference of 1 grade between two radiologists, the higher grade was accepted. If the difference was more than 1 grade, a third radiologist was consulted, and the grade concordant with third grade was accepted. We defined radiographic knee OA as having KL grade ≥2 in one or both knees [5]. Participants with radiographic knee OA and concurrent knee pain were defined as symptomatic knee OA [7]. Our definition of radiographic knee OA applied the same criteria as earlier epidemiologic studies [5], [7], [17]. Although prediction for radiographic knee OA with KL grade ≥2 is worthwhile, there is a continuous relationship between severity of knee OA and risk variables such age, BMI, and pain [18–20]. Therefore, we also investigated the prediction models for more severe radiographic knee OA with KL grade ≥3 and ≥4.

### Development of the scoring system

For risk prediction model development, the association between risk factors and radiographic knee OA was examined by multivariable LR [21]. Based on the development dataset (KNHANES V-1), we included a comprehensive list of variables in Table 1 considered to be potentially associated with knee OA in a risk score model. To simplify the risk model, age range was divided into three levels (<60, 60–69, and ≥70 years). BMI range was also divided into three groups by the cut-off value of overweight (≥23 kg/m^2^) and obesity (≥25 kg/m^2^) based on the definition of obesity in the Asian regions [22]. Backward elimination was performed until we reached a final model with significant covariates. We intentionally used only categorized variables for LR to develop a simple scoring system. We developed a scoring system by assigning scores of 0–2 to multiple categories and scores of 0–1 to binary categories. This scoring system, which was calculated by summing up the arbitrary values for each risk factor, has been widely accepted for the prediction of diseases [23], [24].

**Table 1:** Clinical characteristics of participants of the training, internal validation, and external validation groups.

Characteristics	Training group (KNHANES V-1) n = 1777	Internal validation group (KNHANES V-1) n = 888	External validation group (OAI) n = 4731	P*	*P*†
**Demographics**					
Age (years)	63.0 ± 9.0	63.3 ± 9.1	61.2 ± 9.2	0.241	<0.001
Sex, men	829 (46.7)	425 (47.9)	1966 (41.6)	0.565	0.002
Graduated from college	201 (11.3)	96 (10.8)	990 (20.9)	0.744	<0.001
Low household income (<$50K)	1373 (77.3)	672 (75.7)	1716 (36.3)	0.381	<0.001
Alcohol (>1 serving/week)	670 (37.7)	332 (37.4)	2019 (42.7)	0.899	0.003
Current smoker	301 (16.9)	148 (16.7)	322 (6.8)	0.868	<0.001
**Anthropometric features**					
BMI (kg/m^2^)	23.8 ± 3.1	23.8 ± 3.1	28.6 ± 4.8	0.883	<0.001
Waist circumference (cm)	83.2 ± 9.1	83.3 ± 8.9	102.3 ± 12.8	0.838	<0.001
**Physical activities**					
Walking (≥1 hour/day)	322 (18.1)	170 (19.1)	2049 (43.3)	0.525	<0.001
Moderate physical activity (≥1 hour/day)	206 (11.6)	97 (10.9)	604 (12.8)	0.651	0.135
Heavy physical activity (≥1 hour/day)	124 (7.0)	74 (8.3)	761 (16.1)	0.211	<0.001
**Medical history**					
Diabetes mellitus	270 (15.2)	122 (13.7)	358 (7.6)	0.325	<0.001
Hypertension	701 (39.4)	347 (39.1)	715 (15.1)	0.866	<0.001
Knee pain	308 (17.3)	163 (18.4)	2377 (50.2)	0.518	<0.001
Knee stiffness	170 (9.6)	71 (8.0)	1411 (29.8)	0.197	<0.001
**Study outcome variables**					
Radiographic knee OA	660 (37.1)	298 (33.6)	2638 (55.8)	0.072	<0.001
KL grade 2	245 (13.8)	104 (11.7)	837 (17.7)	0.144	<0.001
KL grade 3	296 (16.7)	132 (14.9)	1247 (26.4)	0.240	<0.001
KL grade 4	119 (6.7)	62 (7.0)	554 (11.7)	0.807	<0.001
Symptomatic knee OA	186 (10.5)	99 (11.1)	1462 (30.9)	0.595	<0.001

Table values are given as mean ± standard deviation or number (%) unless otherwise indicated. BMI, body mass index; KL, Kellgren/Lawrence; KNHANES, Korea National Health and Nutrition Examination Survey; OA, osteoarthritis; OAI, Osteoarthritis Initiative.

*P-values represent significant difference between the training and internal validation group, and were obtained by t-test and chi-square test.

^†^*P*-values represent significant difference between the internal and external validation group, and were obtained by t-test and chi-square test.

### Development of the artificial neural network

ANN models were constructed by use of NeuroSolution version 6.0 (NeuroDimension, Gainesville, FL). NeuroSolution is a professional software that simplifies the construction of ANN [25]. This software allowed simultaneous testing of different type of neural networks including generalized regression neural network, multilayer perceptron, probabilistic neural network, radial basis neural network, feed-forward neural network, and support vector machine. To avoid over-fitting, the prediction models were internally validated using cross validation. Performances of the prediction models were monitored during training and cross-validation to obtain optimal algorithm parameters, such as learning rate, momentum, and number of hidden nodes. The ANN construction was accomplished by the training group. In order to establish a simple prediction model, the same predictors selected in the scoring system were adopted to implement the modeling of ANN input layer.

The ANN model was trained with the five-grade scale of radiographic severity (KL grade of 0–4) as an output variable. This training scheme was similar to multivariate linear regression. However, it produced nonlinear regression function which was optimized for prediction for individuals’ KL grades [26]. Such ANN training scheme has been widely used for analysis of polychotomous grade prediction [27], [28]. Finally, the ANN model was used for prediction of four clinical outcomes with different cut-off values. The four primary outcome variables were presence of radiographic knee OA with KL grade ≥2, ≥3, ≥4, and symptomatic knee OA. In order to compare the performance of the ANN model, LR models for each clinical outcome were also constructed using the same training dataset.

### External validation

Performance of the prediction model was evaluated in independent data, the OAI study. The OAI is a multicenter longitudinal cohort study; a prospective natural history study investigating the development and progression of knee OA in men and women ages 45–79 years at enrollment. Annual OAI interviews began in 2004 at 4 clinical sites, Baltimore, Columbus, Pittsburgh, and Pawtucket. The first 2 years of assessments have been completed, and those data have been publicly released [29]. We used version 0.2.2 AllClinical00, which was comprised of demographic, clinical, and knee imaging data. A total of 4731 of 4796 participants underwent both the knee radiographic examination and were eligible for external validation group. In the OAI, osteophyte and joint space narrowing scores were assessed for each knee by trained radiologists according to the OARSI Atlas grades. For our analysis, we computed KL grades for each knee using the equations provided on the OAI website and used the greater one among the right and left KL grades [18]. Due to the different definition of obesity in the non-Asian regions, the scoring system in the OAI adopted the modified cut-off values of overweight (≥25 kg/m^2^) and obesity (≥30 kg/m^2^) [22].

### Statistical analysis

The prediction models were validated in two populations, the KNHANES V-1 (internal validation group) and OAI (external validation group). Area under the curve (AUC) of the receiver operating characteristic (ROC), accuracy, sensitivity, and specificity of the scoring model, LR, and ANN were calculated. We generated the ROC curves and selected cut-off points which maximized Youden's index [30]. Participants above the cut-off points were classified as being at high risk in each prediction model. We used SPSS 18.0 (SPSS Inc., Chicago, IL) for statistical analysis and MedCalc 12.3 (MedCalc, MariaKerke, Belgium) for ROC analysis.

## Results

### Population characteristics

Characteristics of the KNHANES V-1 and OAI are presented in Table 1. Of 2665 participants from the KNHANES V-1, 958 (35.9%) had radiographic knee OA. Among 958 participants, 285 with pain were classified as having symptomatic knee OA. In the OAI, 2638 (55.8%) of 4731 participants had radiographic knee OA. Among 2638 participants, 1462 had symptomatic knee OA. There were significantly different demographic features between the internal validation group (KNHANES V-1) and external validation group (OAI). Especially, the participants in the OAI had higher BMI and waist circumference, were more likely to have knee pain and stiffness, but were less likely to have diabetes mellitus and hypertension than those in the internal validation group.

### Calculation of prediction models

**Fig 2:**
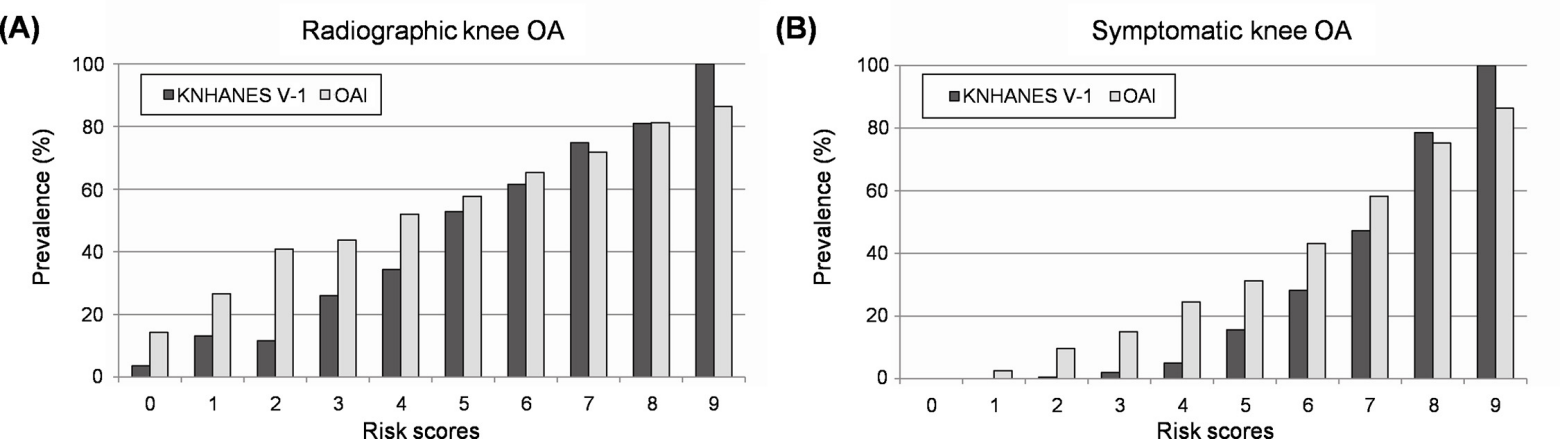
Prevalence of radiographic and symptomatic knee osteoarthritis depending on the risk scores. (A) Prevalence of radiographic knee osteoarthritis. (B) Prevalence of symptomatic knee osteoarthritis. KNHANES, Korea National Health and Nutrition Examination Survey; OA, osteoarthritis; OAI, Osteoarthritis Initiative.

Multivariable LR demonstrated that seven predictors had a statistically significant association with radiographic knee OA in the development dataset (Table 2). The numeric value was assigned to each variable, and we calculated individuals' score (range 0–9). The predictors selected for the scoring system included sex, age, BMI, educational status (graduated from college), hypertension, moderate physical activity, and knee pain. These predictors were also used to establish the LR and ANN models. Fig 2 presents the prevalence of radiographic and symptomatic knee OA for each risk score. In the KNHANES V-1, the prevalence of radiographic knee OA increased gradually, while the prevalence of symptomatic knee OA increased dramatically as the risk scores increased. Consistent results were observed when we applied the scoring system to the OAI. According to ROC analysis, a cut-off of ≥5 was selected for an indicator of high risk group in both the KNHANES V-1 and OAI for all clinical outcomes (S1 Table).

**Table 2:** Multivariate logistic regression analysis for related factors with radiographic knee osteoarthritis in study population, KNHANES V-1.

Variables	no.	β-Coefficient	Odds ratio (95% CI)	*P*	Score assigned
**Sex**					
Male	1254		Reference		
Female	1411	0.43	1.53 (1.27–1.83)	<0.001	1
**Age (years)**					
<60	1163		Reference		
60–69	860	0.83	2.29 (1.86–2.82)	<0.001	1
≥70	642	1.67	5.32 (4.22–6.72)	<0.001	2
**BMI (kg/m**^**2**^**)**					
<23	1080		Reference		
23–24.9	692	0.51	1.67 (1.33–2.08)	<0.001	1
≥25	893	0.96	2.63 (2.13–3.24)	<0.001	2
**Graduated from college**					
Yes	297		Reference		
No	2368	0.41	1.51 (1.09–2.06)	0.011	1
**Hypertension**					
No	1617		Reference		
Yes	1048	0.22	1.25 (1.04–1.49)	0.017	1
**Moderate physical activity (hr/day)**					
<1	2362		Reference		
≥1	303	0.33	1.39 (1.06–1.82)	0.015	1
**Knee pain**					
No	2194		Reference		
Yes	471	0.84	2.31 (1.84–2.89)	<0.001	1

CI, confidence interval; BMI, body mass index.

The ANN was trained with seven predictors, which were selected by the scoring system, as input variables. The model chosen for radiographic knee OA prediction was a multilayer perceptron neural network with back-propagation algorithm [12]. We found three neurons in the hidden layer. When the prediction performance of 10-fold cross validation was assessed in the training group, the final model showed an AUC of 0.80 and an accuracy of 71.9% for radiographic knee OA with KL grade ≥2. This ANN model was superior to the binary ANN models, which were trained separately for each clinical outcome with binary class as an output (S2 Table). Categorization by the binary ANN models caused the loss of the information about severity of knee OA, and it might lead to performance degradation [31].

### Performance of prediction models

The Spearman’s correlations between input variables and KL grade showed low range of 0.05–0.38 in development dataset. The KL grade was more significantly associated with the scoring system (*r* = 0.46, *p*<0.001) and the ANN (*r* = 0.59, *p*<0.001) in the internal validation group. In the external validation group, the scoring system (*r* = 0.26, *p*<0.001) and ANN (*r* = 0.36, *p*<0.001) also showed higher correlation with KL grade than input variables, which showed range of 0.01–0.22.

**Fig 3:**
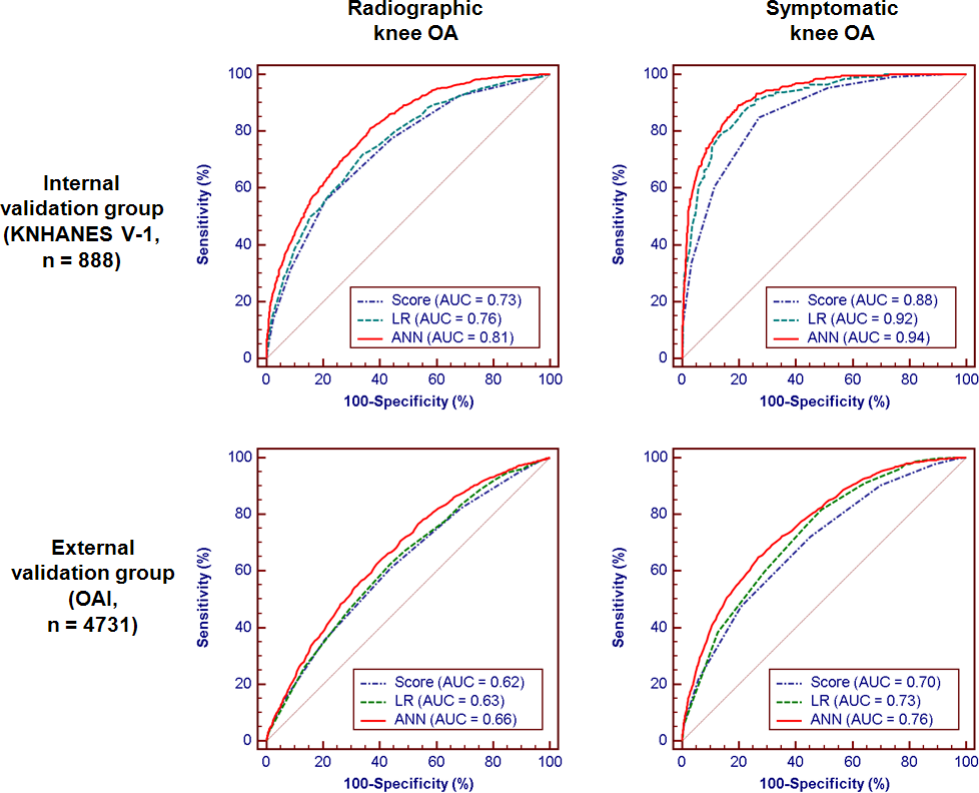
ROC curves for radiographic and symptomatic knee osteoarthritis in internal and external validation groups. ANN, artificial neural network; AUC, area under the receiver operating characteristic curve; KNHANES, Korea National Health and Nutrition Examination Survey; LR, logistic regression; OA, osteoarthritis; OAI, Osteoarthritis Initiative.

Fig 3 shows ROC curves for radiographic and symptomatic knee OA in the internal and external validation groups. In the internal validation, both scoring system and ANN predicted radiographic knee OA (AUC 0.73 versus 0.81, p<0.001) and symptomatic knee OA (AUC 0.88 versus 0.94, p<0.001) with good discriminative ability. When a cut-off of ≥5 was adopted, the scoring system yielded an accuracy of 70.5%, sensitivity of 54.0%, and specificity of 78.8% for radiographic knee OA with KL grade ≥2 in the internal validation. The ANN predicted radiographic knee OA with an accuracy of 73.6%, sensitivity of 73.2%, and specificity of 73.9%, and was significantly superior to the scoring system (*p*<0.001) and LR (*p* = 0.018) in the internal validation group. Both scoring system and ANN showed a lower discriminative ability in predicting radiographic knee OA (AUC 0.62 versus 0.67, p<0.001) and symptomatic knee OA (AUC 0.70 versus 0.76, p<0.001) in the external validation. Table 3 shows the results of prediction modes for 4 clinical outcomes in the internal and external validation groups. We observed increasing prediction performance with increasing KL grade. For example, the AUCs in the internal validation were 0.73, 0.76, and 0.81 for KL grade ≥2, ≥3, and ≥4, respectively.

**Table 3:** Performance of prediction models on internal and external validation groups.

Models	AUC (95% CI)	Accuracy (%) (95% CI)	Sensitivity (%) (95% CI)	Specificity (%) (95% CI)	PPV (%)	NPV (%)
**Internal validation (KNHANES V-1, n = 888)**						
**Radiographic knee OA**						
*KL grade ≥2*						
Score	0.73 (0.69–0.77)	70.5 (67.4–73.5)	54.0 (50.7–57.3)	78.8 (75.9–81.4)	56.3	77.2
LR	0.76 (0.72–0.79)	68.0 (64.8–71.1)	73.8 (70.8–76.7)	65.1 (61.8–68.2)	51.6	83.1
ANN	0.81*^†^ (0.78–0.84)	73.6 (70.6–76.5)	73.2 (70.1–76.0)	73.9 (70.9–76.7)	58.6	84.5
*KL grade ≥3*						
Score	0.76 (0.72–0.80)	74.3 (71.3–77.1)	64.9 (61.7–68.1)	76.9 (74.0–79.7)	44.1	88.7
LR	0.78 (0.75–0.82)	74.5 (71.5–77.4)	68.6 (65.4–71.6)	76.2 (73.3–79.0)	44.6	89.7
ANN	0.85*^†^ (0.82–0.88)	77.7 (74.8–80.4)	77.3 (74.4–80.0)	77.8 (74.9–80.5)	49.3	92.5
*KL grade ≥4*						
Score	0.81 (0.76–0.87)	71.6 (68.5–74.5)	77.4 (74.5–80.1)	71.2 (68.1–74.1)	16.8	97.7
LR	0.84 (0.79–0.88)	75.9 (72.9–78.7)	77.4 (74.5–80.1)	75.8 (72.8–78.5)	19.4	97.8
ANN	0.88* (0.85–0.92)	81.0 (78.2–83.5)	82.3 (79.5–84.7)	80.9 (78.1–83.4)	24.4	98.4
**Symptomatic knee OA**						
Score	0.88 (0.84–0.91)	76.0 (73.0–78.8)	86.9 (84.4–89.0)	74.7 (71.6–77.5)	30.1	97.8
LR	0.92* (0.89–0.94)	88.3 (85.9–90.3)	81.8 (79.1–84.3)	89.1 (86.8–91.0)	48.5	97.5
ANN	0.94* (0.91–0.96)	82.8 (80.1–85.2)	90.9 (88.8–92.7)	81.7 (79.0–84.2)	38.5	98.6
**External validation (OAI, n = 4731)**						
**Radiographic knee OA**						
*KL grade ≥2*						
Score	0.62 (0.60–0.63)	58.8 (57.4–60.2)	60.9 (59.5–62.3)	56.1 (54.7–57.5)	63.6	53.2
LR	0.63 (0.62–0.65)	59.8 (58.3–61.2)	62.5 (61.1–63.9)	56.3 (54.9–57.7)	64.3	54.4
ANN	0.66*^†^ (0.65–0.68)	62.0 (60.6–63.4)	63.3 (61.9–64.7)	60.3 (58.9–61.7)	66.8	56.6
*KL grade ≥3*						
Score	0.62 (0.60–0.63)	56.6 (55.2–58.0)	63.1 (61.7–64.5)	52.6 (51.2–54.0)	45.0	69.9
LR	0.63 (0.62–0.65)	58.1 (56.7–59.5)	66.1 (64.7–67.4)	53.2 (51.7–54.6)	46.4	71.8
ANN	0.68*^†^ (0.66–0.69)	63.8 (62.4–65.2)	59.2 (57.8–60.6)	66.7 (65.3–68.0)	52.2	72.7
*KL grade ≥4*						
Score	0.63 (0.61–0.64)	51.4 (50.0–52.9)	70.6 (69.3–71.9)	48.9 (47.5–50.3)	15.5	92.6
LR	0.67* (0.65–0.68)	63.6 (62.2–65.0)	60.3 (58.9–61.7)	64.0 (62.7–65.4)	18.2	92.4
ANN	0.72*^†^ (0.70–0.73)	65.3 (63.9–66.6)	66.1 (64.7–67.4)	65.2 (63.8–66.5)	20.1	93.5
**Symptomatic knee OA**						
Score	0.70 (0.68–0.71)	60.2 (58.8–61.6)	72.0 (70.6–73.2)	54.9 (53.5–56.3)	41.6	81.4
LR	0.73* (0.71–0.74)	60.5 (59.1–61.9)	81.9 (80.7–83.0)	51.0 (49.5–52.4)	42.8	86.3
ANN	0.76*^†^ (0.75–0.77)	70.6 (69.2–71.8)	64.6 (63.3–66.0)	73.2 (71.9–74.5)	51.9	82.2

ANN, artificial neural networks; AUC, area under the receiver operating characteristic curve; CI, confidence interval; KL, Kellgren/Lawrence; KNHANES, Korea National Health and Nutrition Examination Survey; LR, logistic regression; NPV, negative predictive value; OA, Osteoarthritis; OAI, Osteoarthritis Initiative; PPV, positive predictive value.

*AUC is significantly larger than that of the score model at the level of *p*<0.05.

^†^AUC is significantly larger than that of the LR model at the level of *p*<0.05.

It is important to identify the participants with radiographic knee OA among the participants complaining of knee pain, especially for clinicians [5]. Therefore, we also evaluated the discriminative ability to predict radiographic knee OA in participants with knee pain. Performance of prediction models for radiographic knee OA with KL grade ≥2 among the participants with knee pain is shown in Table 4. The scoring system and ANN showed the similar performance to the results in Table 3 in predicting the internal and external validation subgroups that had knee pain.

**Table 4:** Performance of prediction models for radiographic osteoarthritis among the participants with knee pain.

Models	AUC (95% CI)	Accuracy (%) (95% CI)	Sensitivity (%) (95% CI)	Specificity (%) (95% CI)	PPV (%)	NPV (%)
**Internal validation (KNHANES V-1, n = 163)**						
Score	0.73 (0.65–0.81)	68.1 (60.3–75.1)	68.7 (60.9–75.6)	67.2 (59.3–74.2)	76.4	58.1
LR	0.78 (0.70–0.85)	70.0 (62.2–76.8)	72.7 (65.1–79.3)	65.6 (57.7–72.8)	76.6	60.9
ANN	0.83*^†^ (0.77–0.90)	74.2 (66.7–80.7)	66.7 (58.8–73.8)	85.9 (79.4–90.8)	88.0	62.5
**External validation (OAI, n = 2377)**						
Score	0.60 (0.57–0.62)	55.2 (53.2–57.2)	47.5 (45.4–49.5)	67.5 (65.6–69.4)	69.9	44.6
LR	0.61 (0.58–0.63)	59.1 (57.1–61.1)	51.2 (49.2–53.3)	71.6 (69.7–73.4)	74.2	47.9
ANN	0.66*^†^ (0.63–0.68)	62.4 (60.4–64.4)	64.9 (62.9–66.8)	58.4 (56.4–60.4)	71.3	51.1

ANN, artificial neural networks; AUC, area under the receiver operating characteristic curve; CI, confidence interval; KL, Kellgren/Lawrence; KNHANES, Korea National Health and Nutrition Examination Survey; LR, logistic regression; NPV, negative predictive value; OA, Osteoarthritis; OAI, Osteoarthritis Initiative; PPV, positive predictive value.

*AUC is significantly larger than that of the score model at the level of *p*<0.05.

^†^AUC is significantly larger than that of the LR model at the level of *p*<0.05.

### Development of a risk prediction calculator

**Fig 4:**
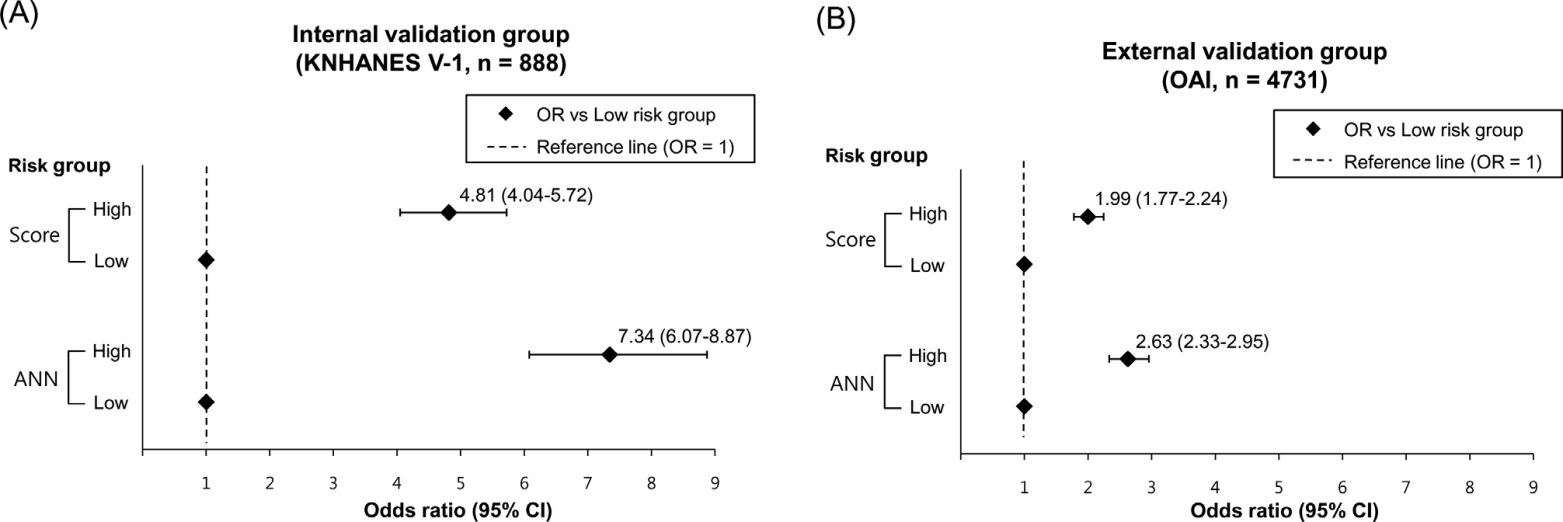
Odds ratios of radiographic knee osteoarthritis in the different risk groups. Comparison of odds ratios of high risk group indicated by the screening score and artificial neural network (A) in internal validation group (KNHANES 2010) and (B) external validation group (OAI). KNHANES, Korea National Health and Nutrition Examination Survey; OR, odds ratio; OAI, Osteoarthritis Initiative.

Risk stratification is important because it provides easier insight into severity [32]. Based on the ROC analysis of prediction models for radiographic knee OA, participants were classified into two group, low risk and high risk groups. In the KNHANES V-1, high risk groups classified by the scoring system and ANN were 33.3% and 43.4% of participants, respectively. In the OAI, high risk groups classified by the scoring system and ANN were 53.4% and 53.5%, respectively. Fig 4 shows odds ratios of radiographic knee OA in the different risk groups indicated by the scoring system and ANN. Although the prediction models for KL grade ≥2 showed the lowest discriminative power, the results demonstrated that the scoring system and ANN effectively predicted the risk for radiographic knee OA with KL grade ≥2. The high risk group defined by the scoring system had odds ratio of 4.81 compared to the low risk group, and the high risk group defined by the ANN had odds ratio of 7.34 in the KNHANES V-1. In the OAI, the odds ratios were lower than those in the KNHANES V-1.

**Fig 5:**
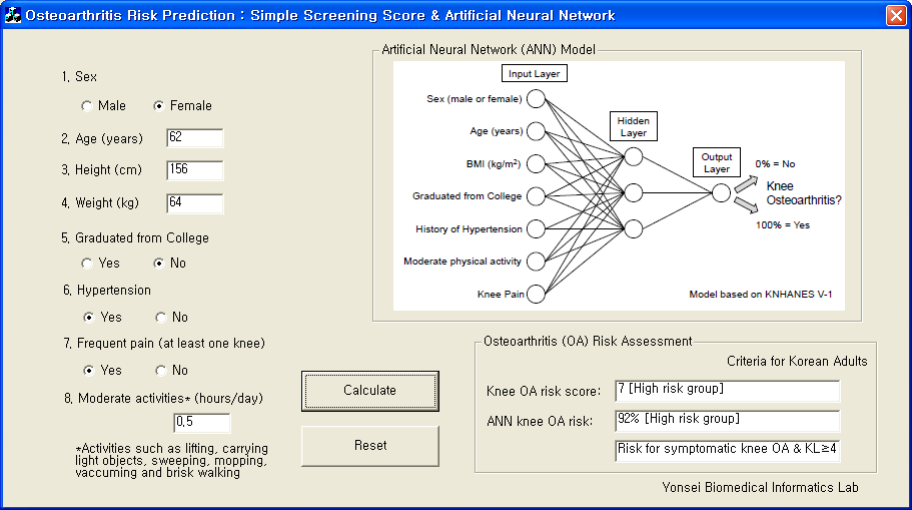
A screen image of the osteoarthritis risk calculator based on artificial neural network. This software is available at https://sites.google.com/site/taekeunyoo/oa-risk-calculator.

We developed a simple ANN calculator to simply measure the knee OA risk. This program is based on Visual C++ computer language. This calculator is designed for use of the self-assessment setting to predict an individual’s risk group. Fig 5 shows a screen image of the ANN calculator.

## Discussion

To our knowledge, this is the first study to develop a simple scoring system and an ANN model for knee OA risk prediction using large population-based data. This self-assessment scoring system may be useful for identifying patients at high risk for knee OA. We found that the performance of the scoring system was improved significantly by the ANN when the same information was given. The predictors including sex, age, BMI, educational status, hypertension, moderate physical activity, and knee pain can be self-assessed or easily identified by the public health center. Such scoring system and ANN might be cost-effective screening tools identifying patients with untreated knee OA. These patients can then be received further evaluation such as knee radiograph and physical examination. However, these were designed for prediction of the disease, therefore they should be used for the purpose of the screening not the clinical diagnosis [7], [33].

The scoring system was developed to be easy and convenient for laypersons to perform a self assessment of knee OA risk. We intended to establish the simplest form of this scoring system. This scoring system may be also applied to mass screening for knee OA or public education about knee OA. If it is possible to use a computer, the ANN calculator could provide not only the risk score but also more accurate result computed by the ANN. Compared to other studies on risk prediction for knee OA, our scoring system and ANN had better performance. According to the Nottingham study, which suggested the first risk prediction model using conventional risk factors, the AUCs of radiographic and symptomatic knee OA were the same value of 0.60 when their prediction model was applied to the OAI population [7]. Our scoring system predicted radiographic and symptomatic knee OA with the AUCs of 0.62 and 0.70, respectively, and the ANN predicted with the AUCs of 0.66 and 0.76, respectively, for the OAI population. In addition, among the participants with pain, the scoring system and ANN predicted radiographic knee OA with the consistently good discriminative ability. If our prediction models retain good performance after validation for the patients complaining of knee pain in the outpatient clinic, it will be possible to use our prediction models as a cost-effective screening tool to determine candidates for knee radiograph.

We suggested that it would be possible to develop a predictive instrument using machine learning techniques such as ANN. The internal and external validation using ROC analysis supported that the ANN had a statistically significant improvement in predicting knee OA. ANN was more effective in analyzing the epidemiological underlying patterns of knee OA compared with the other methods, the scoring system and LR. This finding is consistent with the previous studies on the comparison of ANN and conventional methods in various complicated problems for predicting diseases [12], [25], [34]. Since ANN had an ability to incorporate nonlinearity in high dimensional space, it was possible to consider all factors for the improvement of sensitivity and specificity in predicting [10]. However, several studies pointed out that ANN could be considered as a black box due to its complexity [25]. Moreover, using the gradient descent learning algorithm, ANN intends to converge to local minima [35]. As a result, it suffers from the over-fitting problem. To avoid the local minima, finding optimal parameter is important but it is difficult [35]. Despite the high performance, ANN is mathematically difficult to apply, and this limits acceptance for many clinicians. To overcome this problem, we developed a practicable ANN calculator which can be easily adapted to the users. A major problem with the previous prediction system for knee OA was also difficulty in calculation of LR model [7]. However, the ANN calculator will make it easy to use for the laypersons or clinicians and provide better performance for predicting knee OA.

Similar to earlier studies concerning prediction for knee OA, knee pain was selected as an important predictor [6]. Pain in OA patients is a leading cause of disability and the most common reason for total joint replacement surgery. However, pain is related to a subjective experience and influenced by social and environmental factors. Knee radiography is used as gold standard for knee OA because it reveals objective findings related to clinical outcomes [19], [26]. Even if a patient had radiographic knee OA without pain, recent researches recommended early treatment to prevent development of symptoms [3]. Therefore, both radiographic and symptomatic knee OA should be important clinical outcomes and we evaluated the combination of risk factors for prediction of both knee OA.

Our prediction model included traditional risk variables such as female, age, obesity, educational status. Educational level has been reported to be associated with physical factors on work-related musculoskeletal disorders [36]. Our results suggest that hypertension was associated with knee OA, and it was an unexpected predictor. The role of metabolic syndrome such as hypertension, diabetes mellitus, and hyperlipidemia was unclear [37]. However, recent studies supported the importance of the systemic metabolic effects in the pathophysiology of knee OA [16], and suggested that prevention of metabolic syndrome may reduce knee OA risk [38]. Several traditional risk variables were not included in our prediction models. Knee injury and family history of knee OA were excluded because they were not surveyed in KNHANES V-1. The percent of interim knee injury in the Framingham knee OA study were 2.7%, and odds ratio of interim knee injury with knee OA was 1.8 but it was not significant [39]. In meta-analysis of observational studies, prior history of knee injury was a strong risk factor for the development of knee OA, and odds ratio for case-control studies was 5.34 (95% CI 3.16–9.02) while that for cohort and cross-sectional studies were 3.74 (95% CI 2.16–6.47) and 3.34 (95% CI 1.95–5.75), respectively [40]. According to the previous studies, there is no effect of moderate physical activity on knee OA when the risk model was adjusted for knee injury [41]. Moreover, a prospective cohort study demonstrated an association between greater daily time spent in light intensity physical activities and reduced risk of onset and progression of disability in adults with OA of the knee or risk factors for knee OA [42]. Since we did not adjust for knee injury and this study was based on a cross-sectional survey, it is difficult to determine that moderate physical activity which was significantly associated with knee OA in this study could be direct risk factor for knee OA.

We found the differences in prediction performance between the KNHANES V-1 and OAI. This finding might result from the ethnic difference and genetic background [43]. The two population data have significant demographic and environmental differences influencing the onset and progression of knee OA. A previous study indicated that the undervalued performance was caused by the discrepancy of knee radiograph protocol [18]. While KL grades were directly obtained by two or three radiologists in the KNHANES V-1, the OAI employed OARSI Atlas grades instead of KL grades. Therefore, we needed to compute each KL grade with osteophyte and joint space narrowing score for the OAI. The different type of reading (original KL grade versus calculated KL grade) might affect the performance of the prediction models.

Tam et. al. investigated prediction protocol for predicting knee OA rehabilitation outcome using ANN [44]. To select a treatment protocol for the best improvements according to clinical conditions of patient, they applied the ANN to develop a computerized prediction system. There was a significant correlation between the rankings of the observed and expected pain improvement in the study, and the Spearman's rho was 0.424, which is statistically significant at p < 0.001 [44]. Lusina et. al. have developed an Osteoarthritis Risk Calculator (OA Risk C) and illustrated its acceptability and feasibility in a pilot study of 45 subjects using the Osteoarthritis Policy (OAPol) Model, which is a validated, state-transition simulation of the natural history and management of knee OA [45]. The model included age, sex, race/ethnicity, obesity status, family history of knee OA, occupational exposure to OA risk, and history of knee injury. Eighty-four percent of pilot study participants reported that OA Risk C was easy to understand, and 89% agreed that the graphs depicting their risk were clear and comprehensible [45]. Kerkhof et. al. investigated different types of risk prediction models for incident knee OA with questionnaire/easily obtainable variables, imaging variables, genetic and biochemical markers [46]. The performance of the model with gender, age, BMI, questionnaire variables, and genetic risk score in internal (Rotterdam Study-I), external (Rotterdam Study-II), and external (Chingford study) sets were 0.67, 0.62, and 0.64 of AUC, respectively. The AUC of ANN for KL ≥ 2, ≥ 3, and ≥ 4 was 0.66, 0.68, and 0.72, respectively in our external set. This indicates that our model shows slightly better AUC than the Kerkhof’s model with genetic variables. The study note that a genetic risk score is not a very good predictor of future radiographic knee OA in an elderly population [46].

There are several limitations to this study. First, the study was based on a cross-sectional survey which had several defects due to medical views. For example, the prevalence of disease was based on a health interview survey taken on one occasion. BMI, physical activity status, as well as knee pain could differ according to the time of measurement. Secondly, we did not distinguish between tibiofemoral and patellofemoral knee OA. In recent years, two knee OA subsets have shown different pattern of etiology, risk factors, and symptoms [47]. In this study, KL grade did not consider the difference of these knee OA subsets. Third, the predictors in our prediction models included knee pain which is an important diagnostic criterion of symptomatic knee OA. In previous studies, it was a matter of the researcher's design whether pain was a risk factor or a clinical outcome [5], [7], [19]. Nonetheless, our study is worthwhile. When frequent knee pain occurred, our prediction models for knee OA may provide more accurate decision support than prediction model without knee pain as an input variable. Fourth, our results only apply to subjects not undergoing OA treatment, since we excluded subjects who were receiving treatment for knee OA. It would have been clinically interesting to identify factors associated with these patients with OA treatment in the future study, since they are the most affected clinically.

## Conclusions

In conclusion, the most important finding of this study is the identification of patients at high risk of knee OA who need additional evaluation and appropriate treatment before aggravation. We developed a scoring system and an ANN, and validated them in the large population. The scoring system and ANN can be easily used and might contribute to the advancement of clinical decision tools. Further studies should be targeted at constructing an extended prediction model for progressive knee OA through the collection of prospective data.

## Supporting Information

S1 Table Diagnostic performances at different cut-off values of the scoring system in the KNHANES V-1 and OAI. (DOC)

S2 Table Comparison of the performance using the two training scheme: The ANN trained with 0–4 Kellgren/Lawrence grades versus the ANNs trained with binary class as output variable for each clinical outcome, (DOC)

## References

[1] Peat G, McCarney R, Croft P. Knee pain and osteoarthritis in older adults: a review of community burden and current use of primary health care. Ann Rheum Dis. 2001; 60: 91–97. 11156538 10.1136/ard.60.2.91 PMC1753462

[2] Richmond J, Hunter D, Irrgang J, Jones MH, Levy B, Marx R, et al Treatment of osteoarthritis of the knee (nonarthroplasty). J Am Acad Orthop Surg. 2009; 17: 591–600. 19726743 10.5435/00124635-200909000-00006 PMC3170838

[3] Chu CR, Williams AA, Coyle CH, Bowers ME. Early diagnosis to enable early treatment of pre-osteoarthritis. Arthritis Res Ther. 2012; 14: 212 10.1186/ar3845 22682469 PMC3446496

[4] Schett G, Zwerina J, Axmann R, Willeit J, Stefan K. Risk prediction for severe osteoarthritis. Ann Rheum Dis. 2010; 69: 1573–1574. 10.1136/ard.2009.123661 20388743

[5] Peat G, Thomas E, Duncan R, Wood L, Wilkie R, Hill J, et al Estimating the probability of radiographic osteoarthritis in the older patient with knee pain. Arthritis Rheum. 2007; 57: 794–802. 17530679 10.1002/art.22785

[6] Morvan J, Roux CH, Fautrel B, Rat AC, Euller-Ziegler L, Loeuille D, et al A case–control study to assess sensitivity and specificity of a questionnaire for the detection of hip and knee osteoarthritis. Arthritis Rheum. 2009; 61: 92–99. 10.1002/art.24079 19116973

[7] Zhang W, McWilliams DF, Ingham SL, Doherty SA, Muthuri S, Muir KR, et al Nottingham knee osteoarthritis risk prediction models. Ann Rheum Dis. 2011; 70: 1599–1604. 10.1136/ard.2011.149807 21613308

[8] Takahashi H, Nakajima M, Ozaki K, Tanaka T, Kamatani N, Ikegawa S. Prediction model for knee osteoarthritis based on genetic and clinical information. Arthritis Res Ther. 2010; 12: R187 10.1186/ar3157 20939878 PMC2991022

[9] Zou J, Han Y, So S-S. Overview of artificial neural networks. Methods Mol Biol. 2008; 458: 15–23. 19065803 10.1007/978-1-60327-101-1_2

[10] Motsinger-Reif AA, Ritchie MD. Neural networks for genetic epidemiology: past, present, and future. BioData Min. 2008; 1: 3 10.1186/1756-0381-1-3 18822147 PMC2553772

[11] Yao W, Chen X, Zhao Y, Van Tooren M. Concurrent Subspace Width Optimization Method for RBF Neural Network Modeling. IEEE Trans Neural Netw Learn Syst. 2012; 23: 247–259. 10.1109/TNNLS.2011.2178560 24808504

[12] Baxt WG. Application of artificial neural networks to clinical medicine. Lancet. 1995; 346: 1135–1138. 7475607 10.1016/s0140-6736(95)91804-3

[13] Eller-Vainicher C, Chiodini I, Santi I, Massarotti M, Pietrogrande L, Cairoli E, et al Recognition of morphometric vertebral fractures by artificial neural networks: analysis from GISMO Lombardia Database. PLoS ONE. 2011; 6: e27277 10.1371/journal.pone.0027277 22076144 PMC3208634

[14] Oh K, Lee J, Lee B, Kweon S, Lee Y, Kim Y. Plan and Operation of the 4th Korea National Health and Nutrition Examination Survey (KNHANES IV). Korean J Epidemiol. 2007; 29: 139–145.

[15] Lee MS, Park SK, Hu H, Lee S. Cadmium exposure and cardiovascular disease in the 2005 Korea National Health and Nutrition Examination Survey. Environ Res. 2011; 111: 171–176. 10.1016/j.envres.2010.10.006 21055738 PMC3683977

[16] Lee S, Kim T-N, Kim S-H. Sarcopenic obesity is more closely associated with knee osteoarthritis than is nonsarcopenic obesity: a cross-sectional study. Arthritis Rheum. 2012; 64: 3947–3954. 10.1002/art.37696 23192792

[17] Kellgren JH, Lawrence JS. Radiological assessment of rheumatoid arthritis. Ann Rheum Dis. 1957; 16: 485–493. 13498603 10.1136/ard.16.4.485 PMC1006994

[18] Reichmann WM, Katz JN, Losina E. Differences in self-reported health in the Osteoarthritis Initiative (OAI) and Third National Health and Nutrition Examination Survey (NHANES-III). PLoS ONE. 2011; 6: e17345 10.1371/journal.pone.0017345 21387001 PMC3046148

[19] Muraki S, Oka H, Akune T, Mabuchi A, En-yo Y, Yoshida M, et al Prevalence of radiographic knee osteoarthritis and its association with knee pain in the elderly of Japanese population-based cohorts: The ROAD study. Osteoarthritis Cartilage. 2009; 17: 1137–1143. 10.1016/j.joca.2009.04.005 19410032

[20] Neogi T, Felson D, Niu J, Nevitt M, Lewis CE, Aliabadi P, et al Association between radiographic features of knee osteoarthritis and pain: results from two cohort studies. BMJ. 2009; 339: b2844 10.1136/bmj.b2844 19700505 PMC2730438

[21] Bang H, Vupputuri S, Shoham DA, Klemmer PJ, Falk RJ, Mazumdar M, et al SCreening for Occult REnal Disease (SCORED): a simple prediction model for chronic kidney disease. Arch Intern Med. 2007; 167: 374–381. 17325299 10.1001/archinte.167.4.374

[22] Misra A, Khurana L. Obesity and the Metabolic Syndrome in Developing Countries. JCEM. 2008; 93: 9–30. 10.1210/jc.2008-1595 18987276

[23] Grobman WA, Stamilio DM. Methods of clinical prediction. Am J Obstet Gynecol. 2006; 194: 888–894. 10.1016/j.ajog.2005.09.002 16522430

[24] Jabbour E, Kantarjian H, O’Brien S, Shan J, Garcia-Manero G, Wierda W, et al Predictive factors for outcome and response in patients treated with second-generation tyrosine kinase inhibitors for chronic myeloid leukemia in chronic phase after imatinib failure. Blood. 2011; 117: 1822–1827. 10.1182/blood-2010-07-293977 21030554 PMC4081281

[25] Heiat A. Comparison of artificial neural network and regression models for estimating software development effort. Inform Software Tech. 2002; 44: 911–922.

[26] Razi MA, Athappilly K. A comparative predictive analysis of neural networks (NNs), nonlinear regression and classification and regression tree (CART) models. Expert Syst Appl. 2005; 29: 65–74.

[27] Christy PS, Tervonen O, Scheithauer BW, Forbes GS. Use of a neural network and a multiple regression model to predict histologic grade of astrocytoma from MRI appearances. Neuroradiology. 1995; 37: 89–93. 7761007 10.1007/BF00588619

[28] Lawrentschuk N, Lockwood G, Davies P, Evans A, Sweet J, Toi A, et al Predicting prostate biopsy outcome: artificial neural networks and polychotomous regression are equivalent models. Int Urol Nephrol. 2011; 43: 23–30. 10.1007/s11255-010-9750-7 20464485

[29] Dunlop DD, Song J, Semanik PA, Sharma L, Chang RW. Physical activity levels and functional performance in the osteoarthritis initiative: a graded relationship. Arthritis Rheum. 2011; 63:127–136. 10.1002/art.27760 20862681 PMC3010474

[30] Fluss R, Faraggi D, Reiser B. Estimation of the Youden Index and its associated cutoff point. Biom J. 2005; 47: 458–472. 16161804 10.1002/bimj.200410135

[31] Kononenko I. Inductive and Bayesian Learning in Medical Diagnosis. Appl Artif Intell. 1993; 7: 317–337.

[32] Cook NR. Statistical evaluation of prognostic versus diagnostic models: beyond the ROC curve. Clin Chem. 2008; 54: 17–23. 18024533 10.1373/clinchem.2007.096529

[33] Zhang W, Doherty M, Peat G, Bierma-Zeinstra MA, Arden NK, Bresnihan B, et al EULAR evidence-based recommendations for the diagnosis of knee osteoarthritis. Ann Rheum Dis. 2010; 69: 483–489. 10.1136/ard.2009.113100 19762361

[34] Ghoshal UC, Das A. Models for prediction of mortality from cirrhosis with special reference to artificial neural network: a critical review. Hepatol Int. 2008; 2: 31–38. 10.1007/s12072-007-9026-1 19669277 PMC2716874

[35] Burse K, Manoria M, Kirar VPS. Improved back propagation algorithm to avoid local minima in multiplicative neuron model In Information Technology and Mobile Communication. Springer Berlin Heidelberg; 2011 pp. 67–73.

[36] Gillen M, Yen IH, Trupin L, Swig L, Rugulies R, Mullen K, et al The association of socioeconomic status and psychosocial and physical workplace factors with musculoskeletal injury in hospital workers. Am J Ind Med. 2007; 50: 245–260. 17311255 10.1002/ajim.20429

[37] Hart DJ, Doyle DV, Spector TD. Association between metabolic factors and knee osteoarthritis in women: the Chingford Study. J Rheumatol. 1995; 22: 1118–1123. 7674240

[38] Yoshimura N, Muraki S, Oka H, Kawaguchi H, Nakamura K, Akune T. Association of knee osteoarthritis with the accumulation of metabolic risk factors such as overweight, hypertension, dyslipidemia, and impaired glucose tolerance in Japanese men and women: the ROAD study. J Rheumatol. 2011; 38: 921–930. 10.3899/jrheum.100569 21324967

[39] Felson DT, Zhang Y, Hannan MT, Naimark A, Weissman B, Aliabadi P, et al Risk factors for incident radiographic knee osteoarthritis in the elderly: the Framingham Study. Arthritis Rheum. 1997; 40: 728–733. 9125257 10.1002/art.1780400420

[40] Muthuri SG, McWilliams DF, Doherty M, Zhang W. History of knee injuries and knee osteoarthritis: a meta-analysis of observational studies. Osteoarthritis Cartilage. 2011; 19: 1286–1293. 10.1016/j.joca.2011.07.015 21884811

[41] McAlindon TE, Wilson PW, Aliabadi P, Weissman B, Felson DT. Level of physical activity and the risk of radiographic and symptomatic knee osteoarthritis in the elderly: the Framingham study. Am J Med. 1999; 106: 151–157. 10230743 10.1016/s0002-9343(98)00413-6

[42] Dunlop DD, Song J, Semanik PA, Sharma L, Bathon JM, Eaton CB, et al Relation of physical activity time to incident disability in community dwelling adults with or at risk of knee arthritis: prospective cohort study. BMJ. 2014; 348: g2472 10.1136/bmj.g2472 24782514 PMC4004786

[43] Yoshida S, Aoyagi K, Felson DT, Aliabadi P, Shindo H, Takemoto T. Comparison of the prevalence of radiographic osteoarthritis of the knee and hand between Japan and the United States. J Rheumatol. 2002; 29: 1454–1458. 12136905

[44] Tam SF, Cheing GL, Hui-Chan CW. Predicting osteoarthritic knee rehabilitation outcome by using a prediction model developed by data mining techniques. Int J Rehabil Res. 2004; 27: 65–69. 15097172 10.1097/00004356-200403000-00009

[45] Losina E, Klara K, Michl GL, Collins JE, Katz JN. Development and feasibility of a personalized, interactive risk calculator for knee osteoarthritis. BMC Musculoskelet Disord. 2015; 16: 312 10.1186/s12891-015-0771-3 26494421 PMC4618755

[46] Kerkhof HJ, Bierma-Zeinstra SM, Arden NK, Metrustry S, Castano-Betancourt M, Hart DJ, et al Prediction model for knee osteoarthritis incidence, including clinical, genetic and biochemical risk factors. Ann Rheum Dis. 2014; 73: 2116–2121. 10.1136/annrheumdis-2013-203620 23962456

[47] Peat G, Duncan RC, Wood LRJ, Thomas E, Muller S. Clinical features of symptomatic patellofemoral joint osteoarthritis. Arthritis Res Ther. 2012; 14: 1–10. 22417687 10.1186/ar3779 PMC3446431

