# Supplementary material for: Simple Scoring System and Artificial Neural Network for Knee Osteoarthritis Risk Prediction: A Cross-Sectional Study
Source: PLoS One. 2016 Feb 9;11(2):e0148724. doi: 10.1371/journal.pone.0148724 (PMC4747508; doi:10.1371/journal.pone.0148724)
Supplement: S1 Table — (DOC) [file pone.0148724.s001.doc]

**S1 Table. Diagnostic performances at different cut-off values of the scoring system in the KNHANES V-1 and OAI.** The bold score cut-offs value represent performance at the best Youden's index.

(A) KNHANES V-1

(i) Radiographic knee Osteoarthritis, Kellgren/Lawrence grade ≥2

| Score criterion | Sensitivity | 95% CI | Specificity | 95% CI | +LR | -LR |
| --- | --- | --- | --- | --- | --- | --- |
| ≥2 | 97.81 | 96.7 - 98.6 | 9.37 | 8.0 - 10.9 | 1.08 | 0.23 |
| ≥3 | 92.59 | 90.7 - 94.2 | 31.75 | 29.5 - 34.0 | 1.36 | 0.23 |
| ≥4 | 77.45 | 74.7 - 80.1 | 55.95 | 53.6 - 58.3 | 1.76 | 0.40 |
| **≥5** | **55.74** | **52.5 - 58.9** | **79.26** | **77.3 - 81.2** | **2.69** | **0.56** |
| ≥6 | 31.32 | 28.4 - 34.4 | 91.51 | 90.1 - 92.8 | 3.69 | 0.75 |
| ≥7 | 13.78 | 11.7 - 16.1 | 97.66 | 96.8 - 98.3 | 5.88 | 0.88 |
| ≥8 | 3.86 | 2.7 - 5.3 | 99.53 | 99.1 - 99.8 | 8.24 | 0.97 |
| ≥9 | 0.31 | 0.06 - 0.9 | 100.00 | 99.8 - 100.0 |  | 1.00 |

(ii) Radiographic knee Osteoarthritis, Kellgren/Lawrence grade ≥3

| Score criterion | Sensitivity | 95% CI | Specificity | 95% CI | +LR | -LR |
| --- | --- | --- | --- | --- | --- | --- |
| ≥2 | 98.19 | 96.8 - 99.1 | 8.27 | 7.1 - 9.5 | 1.07 | 0.22 |
| ≥3 | 93.76 | 91.5 - 95.5 | 27.97 | 26.0 - 30.0 | 1.30 | 0.22 |
| ≥4 | 81.44 | 78.1 - 84.5 | 51.46 | 49.3 - 53.6 | 1.68 | 0.36 |
| **≥5** | **65.35** | **61.4 - 69.1** | **76.17** | **74.3 - 78.0** | **2.74** | **0.45** |
| ≥6 | 40.56 | 36.6 - 44.6 | 90.37 | 89.0 - 91.6 | 4.21 | 0.66 |
| ≥7 | 19.21 | 16.2 - 22.6 | 97.32 | 96.5 - 98.0 | 7.18 | 0.83 |
| ≥8 | 5.75 | 4.0 - 7.9 | 99.51 | 99.1 - 99.8 | 11.82 | 0.95 |
| ≥9 | 0.49 | 0.1 - 1.4 | 100.00 | 99.8 - 100.0 |  | 1.00 |

(iii) Radiographic knee Osteoarthritis, Kellgren/Lawrence grade ≥4

| Score criterion | Sensitivity | 95% CI | Specificity | 95% CI | +LR | -LR |
| --- | --- | --- | --- | --- | --- | --- |
| ≥2 | 99.45 | 97.0 - 100.0 | 7.25 | 6.3 - 8.3 | 1.07 | 0.076 |
| ≥3 | 97.24 | 93.7 - 99.1 | 24.48 | 22.8 - 26.2 | 1.29 | 0.11 |
| ≥4 | 90.61 | 85.4 - 94.4 | 46.46 | 44.5 - 48.4 | 1.69 | 0.20 |
| **≥5** | **79.56** | **72.9 - 85.2** | **70.05** | **68.2 - 71.8** | **2.66** | **0.29** |
| ≥6 | 61.88 | 54.4 - 69.0 | 86.59 | 85.2 - 87.9 | 4.62 | 0.44 |
| ≥7 | 33.15 | 26.3 - 40.5 | 95.49 | 94.6 - 96.3 | 7.35 | 0.70 |
| ≥8 | 12.15 | 7.8 - 17.8 | 99.07 | 98.6 - 99.4 | 13.13 | 0.89 |
| ≥9 | 1.10 | 0.1 - 3.9 | 99.96 | 99.8 - 100.0 | 27.45 | 0.99 |

(iv) Symptomatic knee Osteoarthritis

| Score criterion | Sensitivity | 95% CI | Specificity | 95% CI | +LR | -LR |
| --- | --- | --- | --- | --- | --- | --- |
| ≥2 | 100.00 | 98.7 - 100.0 | 7.61 | 6.6 - 8.7 | 1.08 | 0.00 |
| ≥3 | 99.30 | 97.5 - 99.9 | 25.67 | 23.9 - 27.5 | 1.34 | 0.027 |
| ≥4 | 95.44 | 92.3 - 97.5 | 48.66 | 46.6 - 50.7 | 1.86 | 0.094 |
| **≥5** | **84.91** | **80.2 - 88.9** | **72.86** | **71.0 - 74.6** | **3.13** | **0.21** |
| ≥6 | 60.70 | 54.8 - 66.4 | 88.57 | 87.2 - 89.8 | 5.31 | 0.44 |
| ≥7 | 33.68 | 28.2 - 39.5 | 96.81 | 96.0 - 97.5 | 10.55 | 0.69 |
| ≥8 | 12.63 | 9.0 - 17.1 | 99.62 | 99.3 - 99.8 | 33.40 | 0.88 |
| ≥9 | 1.05 | 0.2 - 3.0 | 100.00 | 99.8 - 100.0 |  | 0.99 |

+LR, positive likelihood ratio; -LR, negative likelihood ratio.

(B) Osteoarthritis Initiative

(i) Radiographic knee Osteoarthritis, Kellgren/Lawrence grade ≥2

| Score criterion | Sensitivity | 95% CI | Specificity | 95% CI | +LR | -LR |
| --- | --- | --- | --- | --- | --- | --- |
| ≥2 | 99.16 | 98.7 - 99.5 | 3.06 | 2.4 - 3.9 | 1.02 | 0.27 |
| ≥3 | 94.49 | 93.5 - 95.3 | 11.58 | 10.2 - 13.0 | 1.07 | 0.48 |
| ≥4 | 82.29 | 80.8 - 83.7 | 31.34 | 29.4 - 33.4 | 1.20 | 0.56 |
| **≥5** | **61.06** | **59.2 - 62.9** | **56.03** | **53.9 - 58.2** | **1.39** | **0.70** |
| ≥6 | 35.71 | 33.9 - 37.6 | 79.43 | 77.6 - 81.1 | 1.74 | 0.81 |
| ≥7 | 14.29 | 13.0 - 15.7 | 93.78 | 92.7 - 94.8 | 2.30 | 0.91 |
| ≥8 | 3.84 | 3.1 - 4.6 | 98.95 | 98.4 - 99.3 | 3.65 | 0.97 |
| ≥9 | 0.72 | 0.4 - 1.1 | 99.86 | 99.6 - 100.0 | 5.03 | 0.99 |

(ii) Radiographic knee Osteoarthritis, Kellgren/Lawrence grade ≥3

| Score criterion | Sensitivity | 95% CI | Specificity | 95% CI | +LR | -LR |
| --- | --- | --- | --- | --- | --- | --- |
| ≥2 | 99.28 | 98.8 - 99.6 | 2.50 | 2.0 - 3.1 | 1.02 | 0.29 |
| ≥3 | 95.33 | 94.2 - 96.3 | 10.36 | 9.3 - 11.5 | 1.06 | 0.45 |
| ≥4 | 84.42 | 82.7 - 86.1 | 28.75 | 27.1 - 30.4 | 1.18 | 0.54 |
| **≥5** | **63.27** | **61.0 - 65.5** | **52.51** | **50.7 - 54.3** | **1.33** | **0.70** |
| ≥6 | 38.68 | 36.4 - 41.0 | 76.92 | 75.4 - 78.4 | 1.68 | 0.80 |
| ≥7 | 15.92 | 14.3 - 17.7 | 92.48 | 91.5 - 93.4 | 2.12 | 0.91 |
| ≥8 | 4.06 | 3.2 - 5.1 | 98.29 | 97.8 - 98.7 | 2.38 | 0.98 |
| ≥9 | 0.89 | 0.5 - 1.4 | 99.79 | 99.6 - 99.9 | 4.34 | 0.99 |

(iii) Radiographic knee Osteoarthritis, Kellgren/Lawrence grade ≥4

| Score criterion | Sensitivity | 95% CI | Specificity | 95% CI | +LR | -LR |
| --- | --- | --- | --- | --- | --- | --- |
| ≥2 | 99.10 | 97.9 - 99.7 | 1.94 | 1.5 - 2.4 | 1.01 | 0.47 |
| ≥3 | 96.20 | 94.3 - 97.6 | 8.78 | 7.9 - 9.7 | 1.05 | 0.43 |
| ≥4 | 88.07 | 85.1 - 90.6 | 25.31 | 24.0 - 26.7 | 1.18 | 0.47 |
| **≥5** | **70.71** | **66.7 - 74.5** | **48.79** | **47.3 - 50.3** | **1.38** | **0.60** |
| ≥6 | 43.94 | 39.8 - 48.2 | 72.97 | 71.6 - 74.3 | 1.63 | 0.77 |
| ≥7 | 19.17 | 16.0 - 22.7 | 90.41 | 89.5 - 91.3 | 2.00 | 0.89 |
| ≥8 | 5.42 | 3.7 - 7.7 | 97.77 | 97.3 - 98.2 | 2.43 | 0.97 |
| ≥9 | 1.63 | 0.7 - 3.1 | 99.69 | 99.5 - 99.8 | 5.22 | 0.99 |

(iv) Symptomatic knee Osteoarthritis

| Score criterion | Sensitivity | 95% CI | Specificity | 95% CI | +LR | -LR |
| --- | --- | --- | --- | --- | --- | --- |
| ≥2 | 99.86 | 99.5 - 100.0 | 2.57 | 2.1 - 3.2 | 1.02 | 0.053 |
| ≥3 | 97.87 | 97.0 - 98.5 | 10.90 | 9.9 - 12.0 | 1.10 | 0.20 |
| ≥4 | 90.32 | 88.7 - 91.8 | 30.01 | 28.4 - 31.6 | 1.29 | 0.32 |
| **≥5** | **72.25** | **69.9 - 74.5** | **54.87** | **53.1 - 56.6** | **1.60** | **0.51** |
| ≥6 | 47.46 | 44.9 - 50.1 | 79.21 | 77.8 - 80.6 | 2.28 | 0.66 |
| ≥7 | 21.84 | 19.7 - 24.1 | 94.24 | 93.4 - 95.0 | 3.79 | 0.83 |
| ≥8 | 6.52 | 5.3 - 7.9 | 99.14 | 98.8 - 99.4 | 7.61 | 0.94 |
| ≥9 | 1.30 | 0.8 - 2.0 | 99.91 | 99.7 - 100.0 | 14.21 | 0.99 |

+LR, positive likelihood ratio; -LR, negative likelihood ratio.
