# Supplementary material for: Simple Scoring System and Artificial Neural Network for Knee Osteoarthritis Risk Prediction: A Cross-Sectional Study
Source: PLoS One. 2016 Feb 9;11(2):e0148724. doi: 10.1371/journal.pone.0148724 (PMC4747508; doi:10.1371/journal.pone.0148724)
Supplement: S2 Table — (DOC) [file pone.0148724.s002.doc]

**S2 Table. Comparison of the performance using the two training scheme:** **The ANN trained with 0-4 Kellgren/Lawrence grades versus the ANNs trained with binary class as output variable for each clinical outcome**. (A) The AUCs of the prediction models in internal validation group. (B) The AUCs of the prediction models in external validation group.

(A) Internal validation (KNHANES V-1, n = 888)

|  | **AUC (95% CI)** | |  |
| --- | --- | --- | --- |
|  | **ANN model trained with 0-4 KL grades** | **ANN models trained with binary class for each clinical outcome** | ***P*** |
| **Radiographic knee OA** |  |  |  |
| KL grade ≥2 | **0.81 (0.78-0.84)** | 0.78 (0.74-0.81) | <0.001 |
| KL grade ≥3 | **0.85 (0.82-0.88)** | 0.81 (0.78-0.85) | 0.002 |
| KL grade ≥4 | **0.88 (0.85-0.92)** | 0.86 (0.82-0.90) | 0.018 |
| **Symptomatic knee OA** | **0.94 (0.91-0.96)** | 0.93 (0.91-0.96) | 0.493 |

(B) External validation (Osteoarthritis Initiative, n = 4731)

|  | **AUC (95% CI)** | |  |
| --- | --- | --- | --- |
|  | **ANN model trained with 0-4 KL grades** | **ANN models trained with binary class for each clinical outcome** | ***P*** |
| **Radiographic knee OA** |  |  |  |
| KL grade ≥2 | **0.66 (0.65-0.68)** | 0.65 (0.64-0.67) | 0.075 |
| KL grade ≥3 | **0.68 (0.66-0.69)** | 0.65 (0.64-0.67) | <0.001 |
| KL grade ≥4 | **0.72 (0.70-0.73)** | 0.68 (0.65-0.70) | <0.001 |
| **Symptomatic knee OA** | 0.76 (0.75-0.77) | **0.76 (0.75-0.78)** | 0.732 |
